# Contrast-enhanced ultrasound in the evaluation and management of solid renal lesions based on EFSUMB guidelines

**DOI:** 10.1007/s10396-025-01546-z

**Published:** 2025-04-25

**Authors:** Laura Eusebi, Federica Masino, Michele Bertolotto, Manuela Montatore, Giuseppe Sortino, Lucia Pitoni, Stefano Santarelli, Andrea Benedetto Galosi, Giuseppe Guglielmi

**Affiliations:** 1 Department of Clinical and Experimental Medicine, Foggia University School of Medicine, Viale L. Pinto 1, 71121 Foggia, FG Italy; 2 Radiology Unit, “Carlo Urbani” Hospital, Via Aldo Moro 52, 60035 Jesi, AN Italy; 3 Radiology Unit, “Cattinara” Hospital, Trieste University, Strada Di Fiume 447, 34149 Trieste, TS Italy; 4 Urology Unit, “Carlo Urbani” Hospital, Via Aldo Moro 52, 60035 Jesi, AN Italy; 5 Nephrology Unit, “Carlo Urbani” Hospital, Via Aldo Moro 52, 60035 Jesi, AN Italy; 6 Urology Unit, “Riuniti Torrette” Hospital Di Ancona, Via Conca 71, 60126 Torrette, AN Italy; 7 Radiology Unit, “Dimiccoli” Hospital, Viale Ippocrate 15, 70051 Barletta, BT Italy; 8 Radiology Unit, IRCCS Casa Sollievo Della Sofferenza” Hospital, Viale Cappuccini 1, San Giovanni Rotondo, 71013 Foggia, Italy

**Keywords:** CEUS, Contrast-enhanced ultrasound, Diagnostic imaging, Renal mass, Renal solid lesion

## Abstract

Contrast-enhanced ultrasound (CEUS) has become an important tool for the detection and characterization of solid renal lesions, complementing traditional B-mode US, which often struggles to distinguish between solid and cystic masses. CEUS enhances diagnostic accuracy by providing detailed vascularization information, helping to identify potentially malignant solid lesions and differentiate between pseudotumours and solid masses. Moreover, CEUS employs a contrast agent that is safe even for nephropathic patients and can be used without concern for kidney function, making it an advantageous method for renal imaging. In this narrative review, following the European Federation of Societies for Ultrasound in Medicine and Biology guidelines, the authors show its main indications: characterizing indeterminate renal lesions in patients with transplanted kidney, monitoring non-surgical lesions, diagnosing inflammatory-infectious renal pathologies, and assessing advanced renal lesions and masses in the surgical bed.

## Introduction

### Basics of CEUS

**C**EUS is widely available as it only requires a US machine, and it is relatively inexpensive as only a few milliliters of contrast medium are used. Additionally, it is a safe method that can be used in nephropathic patients, regardless of creatinine levels, as the contrast medium is eliminated via the lungs [1–3]. CEUS also reduces the interobserver variability typical of US. Time-intensity curves allow for a quantitative assessment that, in the future, could help predict the biological behaviour of some renal lesions. Due to these positive characteristics, CEUS is mentioned in the European Association of Urology (EAU) guidelines with sensitivity, specificity, and negative predictive values approaching 100% [1, 4].

CEUS uses a contrast medium consisting of sulfur hexafluoride microbubbles stabilized by an external phospholipid shell. These microbubbles have a diameter smaller than 10 μm, so when injected intravenously, they pass through the pulmonary circulation without any problems. However, their size does not allow them to pass through the endothelium, so they are classified as blood-pool contrast agents. Under normal conditions, they do not escape the capillaries, meaning there is no extravascular phase, which causes a loss of information regarding capillary permeability and no urographic information, unlike computed tomography (CT) or magnetic resonance imaging (MRI) contrast agents. However, CEUS is extremely sensitive in studying microcirculation as the contrast medium remains confined to the blood vessels, combined with the maximum spatial resolution typical of US exams. In addition to qualitative evaluation of local perfusion, colorimetric maps can be created to assess microcirculation density, and time-intensity signal curves can be produced. The dynamics of renal contrast enhancement on CEUS are similar to what can be achieved with CT and MRI, except for the excretory urographic phase [5, 6]. The modality's limitations are the same as those of US: acoustic shadowing behind calcifications, acoustic shadowing behind intestinal gas, and beam attenuation in the case of deep lesions and patients with a high body mass index. There are also regulatory limitations as renal CEUS studies are currently off-label despite clinical evidence. Nonetheless, the European Federation of Societies for Ultrasound in Medicine and Biology (EFSUMB) has published specific guidelines for the use of CEUS in the uro-nephrological field. US contrast agents are physiologically safe (no need for preliminary laboratory tests, even in patients with liver disease or renal insufficiency). The literature reports a very low incidence of severe anaphylactoid reactions (0.001%). As mentioned, the components of US contrast agents (phospholipids) are eliminated by the liver in very small quantities (on average, 1.2–2.4 ml of contrast is injected), while sulfur hexafluoride is eliminated through respiration. There have been no fatal events in a series of 23,000 patients. These compounds are stable enough to last for the duration of a normal US exam (three to five minutes) [5].

The exam is usually performed with the patient in the supine or oblique position, elevating the side under examination. If there is meteorism of the colic flexures, a posterior approach with the probe placed at the level of the posterior axillary line or even more dorsally may be necessary. In these cases, the subcostal approach is not possible, and the study of the kidney must be done using the intercostal window, which does not allow visualization of the entire kidney in a single scan. In children and lean individuals, the prone position may be used due to the reduced thickness of the dorsal paravertebral muscles. If one or both kidneys are not identifiable in their normal location, the scan should be extended to the entire pelvis to search for ectopic and/or dysmorphic kidneys (presacral ectopia, horseshoe kidney, crossed ectopia). To determine the longitudinal diameter of the kidney, the best scanning plane should be found by moving the probe in real time so that both poles are well represented and the parenchymal thickness is uniform [7].

### Principles of application of CEUS in the evaluation of renal lesions

The characterization of renal lesions is a critical aspect of diagnostic imaging as it directly impacts clinical decision-making and patient management. Incidental renal lesions are frequently encountered in clinical practice, with US being the primary detection method, identifying 83% of cases compared to 15% for CT. However, B-mode US has significant limitations, particularly in distinguishing between solid and cystic lesions [8]. This distinction is essential as vascularized solid lesions are more likely to be malignant, necessitating further evaluation and intervention. Despite the widespread use of US and its availability, traditional US techniques, including color-Doppler, have been deemed insufficiently sensitive and specific by major guidelines. This has led to a growing interest in CEUS as a complementary and potentially superior imaging modality. Given the increasing clinical reliance on CEUS and evolving guideline recommendations, this review aims to provide a comprehensive analysis of its role in solid renal lesion assessment, highlighting its advantages, limitations, and future applications.

The American College of Radiology (ACR) as well as the EAU mentioned CEUS as a method that can be used with the same appropriateness as contrast-enhanced CT or MRI. CEUS helps overcome the main limitation of US, i.e., the lack of contrast medium, while also addressing the inherent limitations of the method, such as low accuracy and lack of specificity. CEUS also represents a valid alternative for better characterizing lesions that present pseudo-enhancement on CT, and consequently indeterminate lesions.

### Ceus indications in renal imaging according to EFSUMB guidelines

#### Characterization of Indeterminate Renal Lesions

CEUS is not only useful for distinguishing between solid and cystic forms but also represents the first question a radiologist should ask when confronted with a newly discovered renal mass. For solid forms, CEUS allows the differentiation between pseudotumours (e.g., anatomical variants) and tumours (newly formed lesions) with higher sensitivity and specificity than CT (CEUS: SE 91%, SP 76%; CECT SE 85%, SP 64%) [3, 9] (Table 1).

**Table 1:** Comparison of renal pseudotumors and tumours, including their ultrasound (US) and contrast-enhanced ultrasound (CEUS) features. *AVM* arteriovenous malformation, *RMC* renal medullary carcinoma, *SRCC* sarcomatoid renal cell carcinoma

Lesion		US features	CEUS feature
Pseudotumours	Prominent Column of Bertin	Isoechoic to renal cortex, smoothly blending with surrounding parenchyma, typically between pyramids	No enhancement difference from adjacent cortex
	Persistent Fetal Lobulation	Lobulated renal contour with indentations between pyramids, no mass effect	Normal enhancement pattern like renal cortex
	Dromedary Hump	Cortical bulge (usually left kidney), iso- or slightly hypoechoic to adjacent cortex	Homogeneous enhancement like adjacent cortex
	Renal Incision (post-surgical change)	Hyperechoic linear area with or without shadowing, no significant mass effect	Follows surrounding parenchymal enhancement pattern
	Infectious Renal Pseudotumors (e.g., Focal Pyelonephritis, Abscess)	Hypoechoic, ill-defined lesion, no clear mass effect, possible increased vascularity in pyelonephritis, abscess may show debris	Pyelonephritis: Delayed or decreased enhancement. Abscess: Non-enhancing central area with peripheral rim enhancement
	Vascular Renal Pseudotumors (e.g., AVM, Infarct)	AVM: Hypoechoic with color Doppler flow. Infarct: Hypoechoic, wedge-shaped	AVM: Rapid wash-in and washout, early draining vein. Infarct: Non-enhancing avascular area
Tumours	Renal Cell Carcinoma	Solid, hypoechoic; or isoechoic; irregular borders; increased vascularity	Early arterial hyperenhancement, washout in venous phase, heterogeneous enhancement
	Angiomyolipoma	Highly echogenic lesion (due to fat), no shadowing, can have cystic areas	Variable enhancement, may be heterogeneous; lipid-poor AMLs can mimic RCC
	Oncocytoma	Solid, well-defined, central scar possible (but not always seen)	Strong arterial enhancement, no significant washout, may have central non-enhancing scar
	Transitional Cell Carcinoma	Hypoechoic, centrally located, often in renal pelvis, may cause hydronephrosis	Mild to moderate early enhancement, rapid washout
	Rare Tumours (RMC, SRCC)	**Renal Medullary Carcinoma (RMC):** Poorly defined, infiltrative, affects young patients with sickle cell trait. **Signet Ring Cell Carcinoma (SRCC):** Aggressive, hypoechoic mass, infiltrative	**RMC:** Rapid wash-in, heterogeneous enhancement with necrotic areas. **SRCC:** Heterogeneous enhancement, early washout, aggressive behavior

**Fig. 1:**
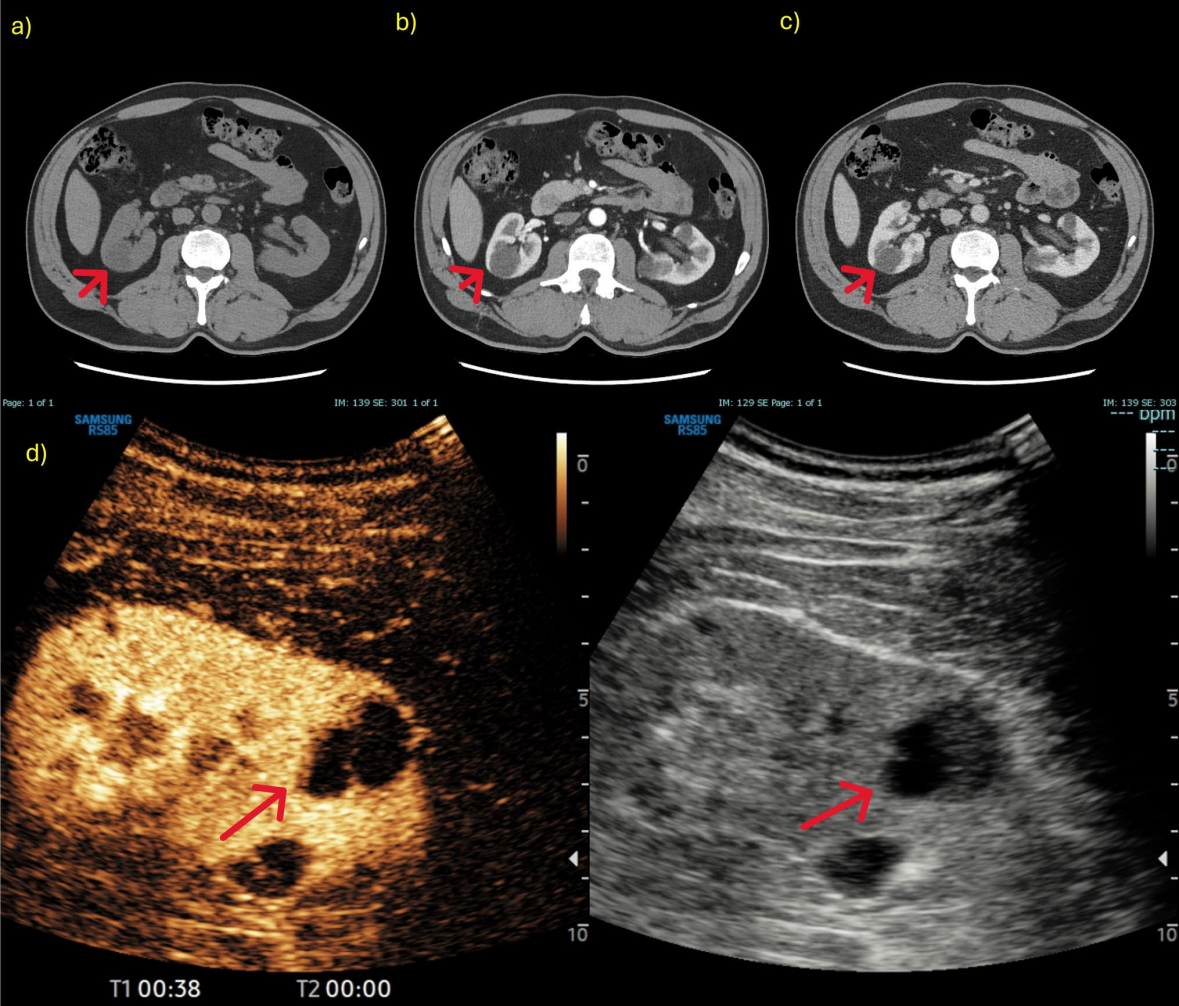
**a-d** CT images of a right renal lesion (red arrow) that appeared hypodense in the basal phase (**a**) but seemed to show a pseudo-enhancement in the parenchymal (**b**) and nephrographic (**c**) phases. CEUS evaluation (**d**) confirmed a non-contrast enhancing lesion; US images were obtained with the patient lying supine

**Fig. 2:**
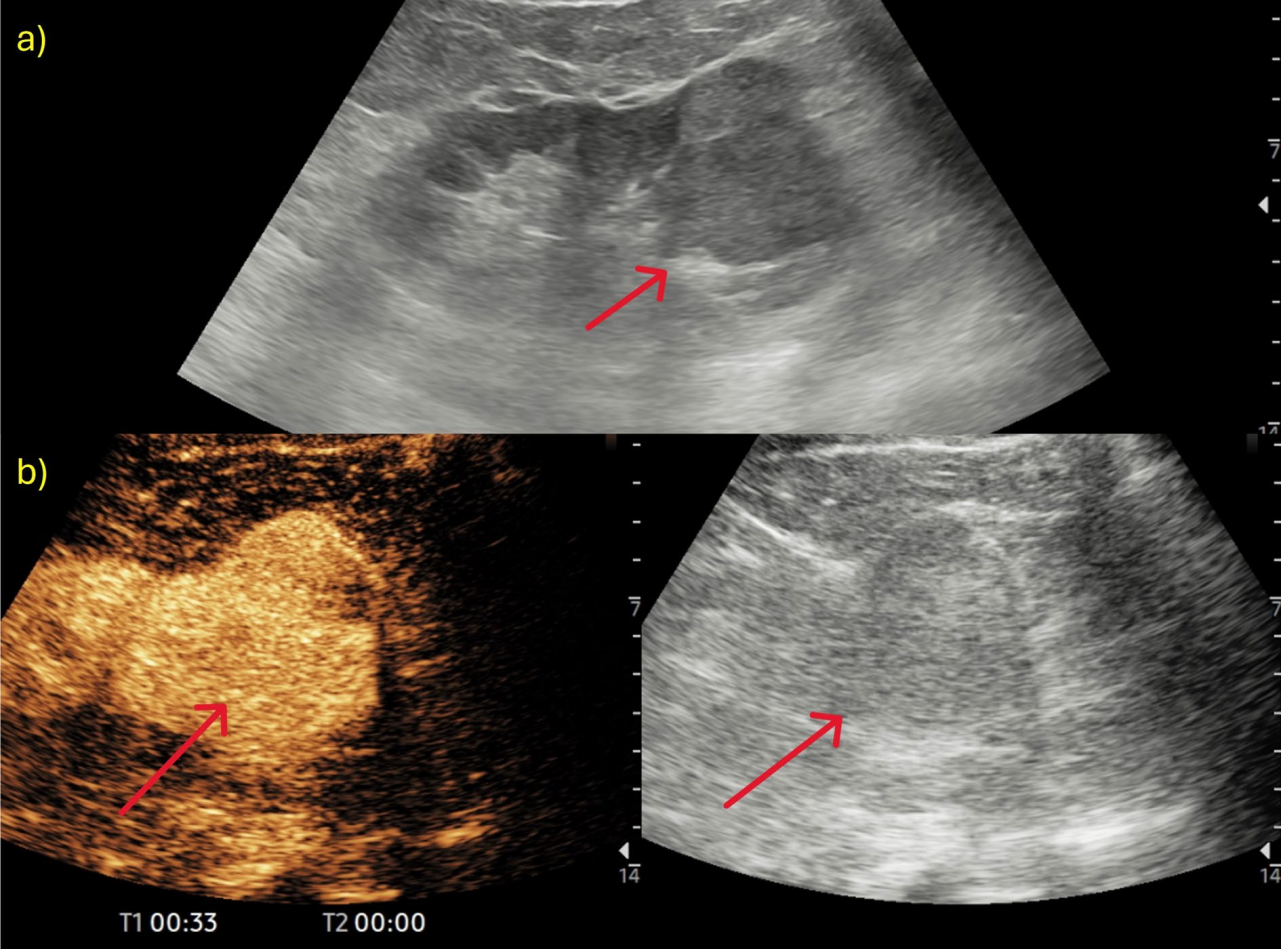
**a, b** A case of indeterminate lesion. US images of a renal evaluation in B-mode (**a**) showed a hypo-isoechoic formation (red arrow) in the right renal lower pole, confirmed on CEUS (**b**) as a renal mass with homogeneous contrast enhancement; US images were obtained with the patient lying supine

**Fig. 3:**
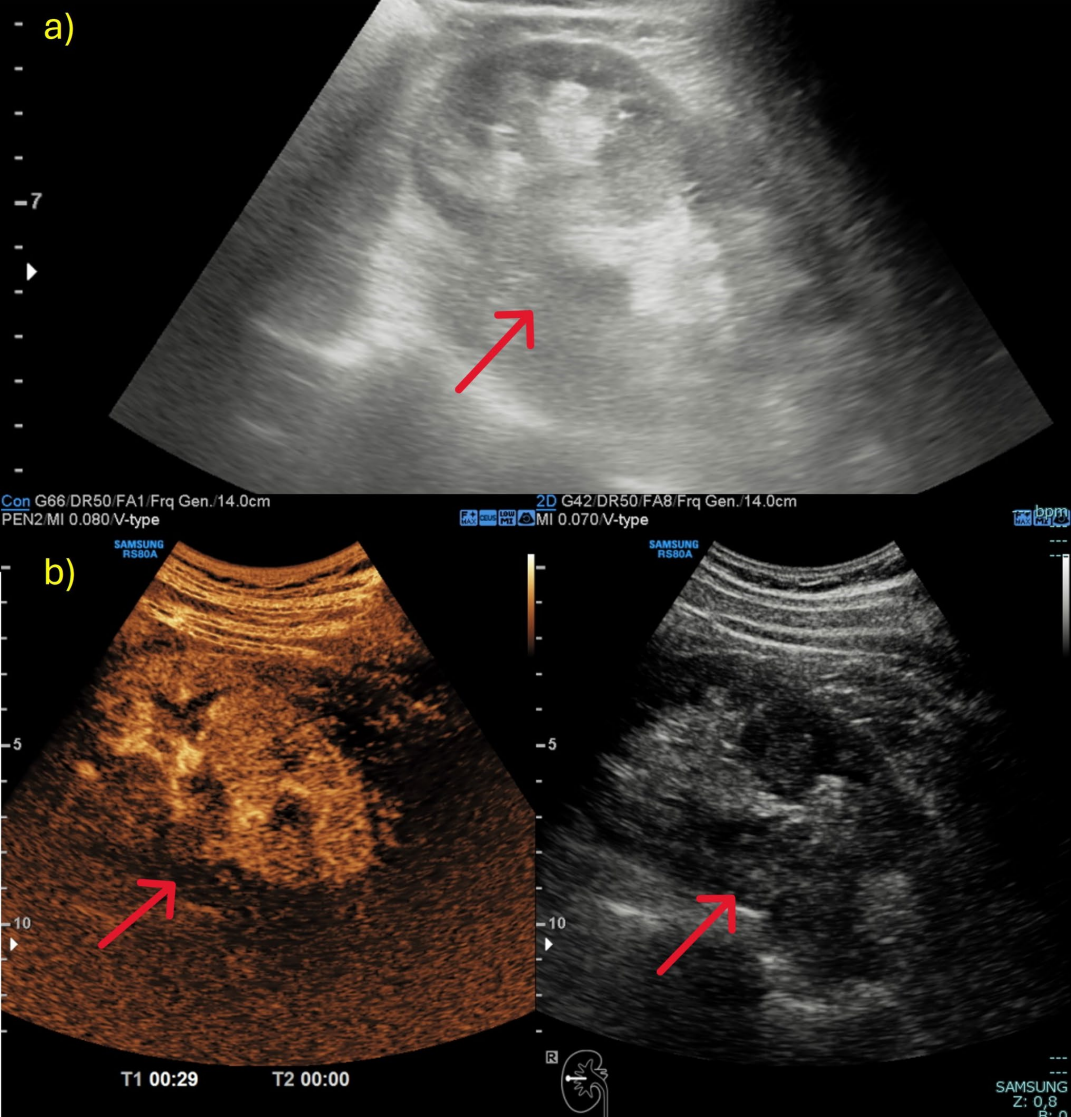
**a, b** A case of pseudotumour. US images of a renal evaluation in B-mode (**a**) showed a bumpy and pseudo-nodular appearance of the cortical region of the renal middle third. CEUS examination (**b**) revealed no pathological enhancement with findings attributable to a prominent column of Bertin. US images were obtained with the patient lying supine

**Fig. 4:**
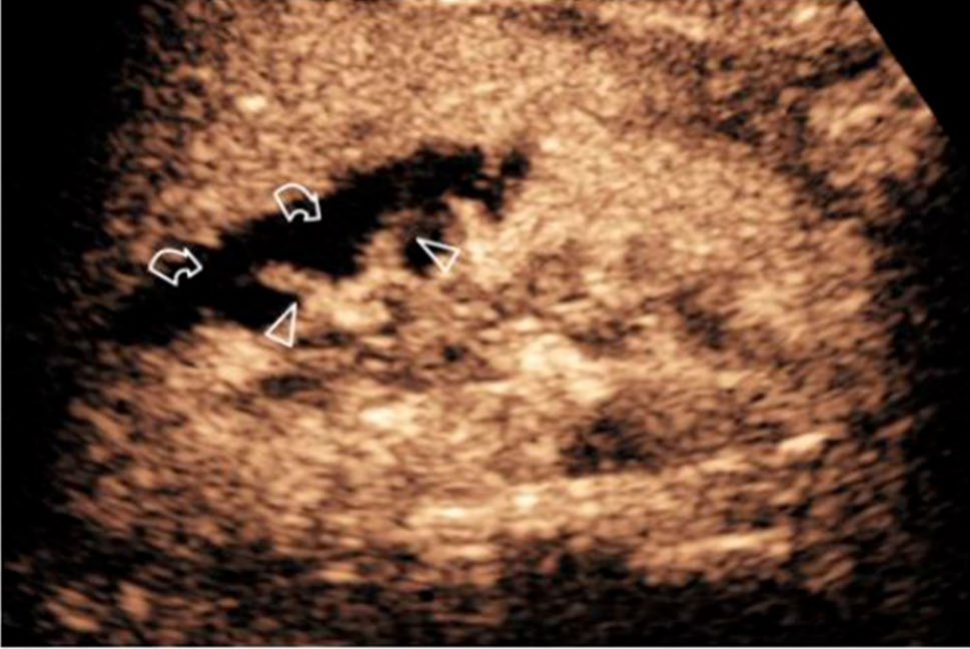
A case of a vascular lesion. CEUS showed focal acute cortical ischemia (curved arrows) and patent interlobar vessels (arrowheads). *Image source: Granata A, Campo I, Lentini P, Pesce F, Gesualdo L, Basile A, et al. Role of Contrast-Enhanced Ultrasound (CEUS) in Native Kidney Pathology: Limits and Fields of Action. Diagnostics. 2021 Jun 8;11(6):1058. This is an open access article distributed under the terms of the Creative Commons Attribution (CC BY) 4.0 license. (http://creativecommons.org/licenses/by/4.0/)

**Fig. 5:**
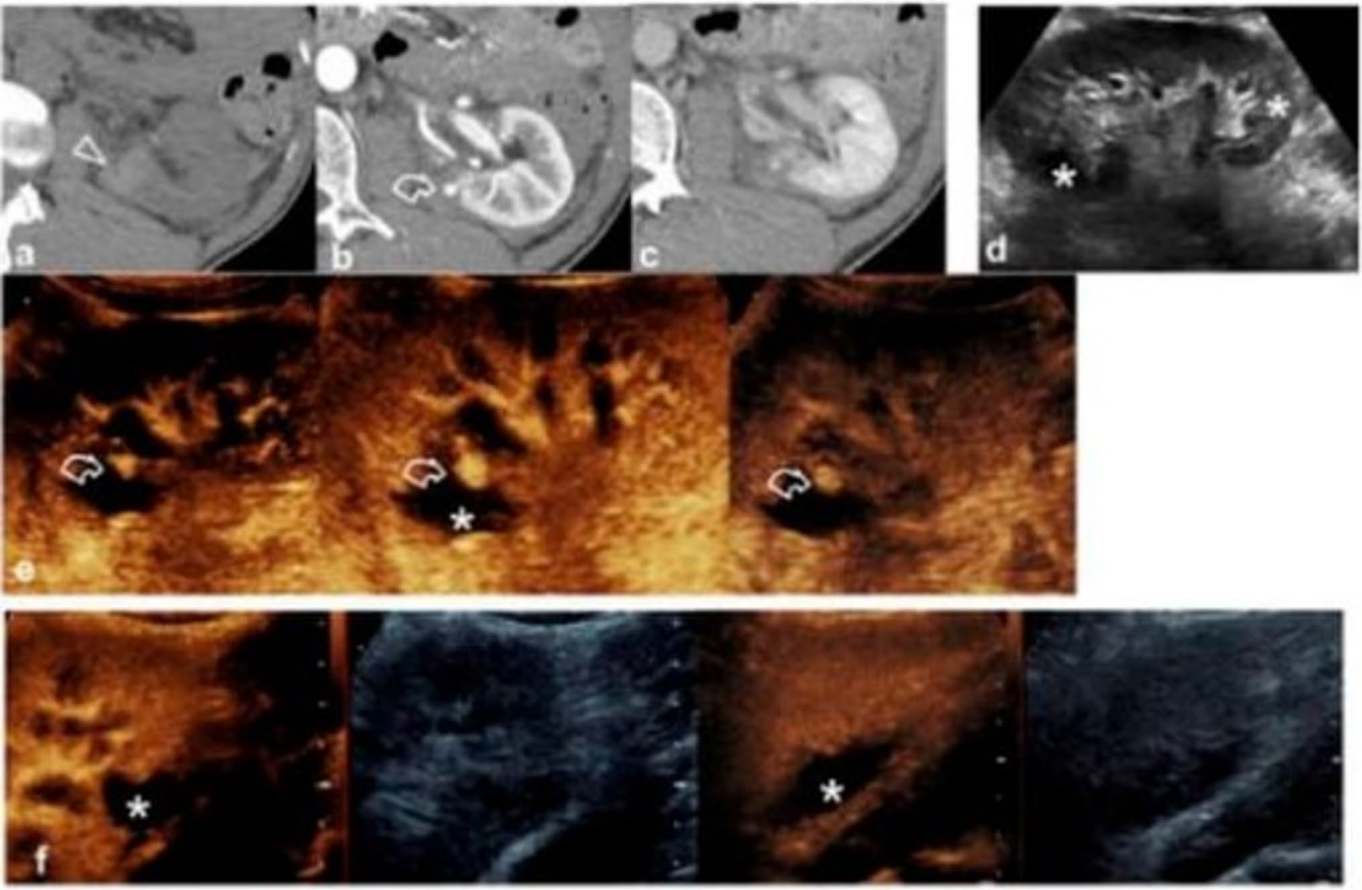
**a-f** A case of a vascular lesion. NECT images (**a**) showed a parenchymal hyper-density from a recent hematoma (arrowhead) at the lower pole of the left kidney. CECT images showed a pseudoaneurysm (curved arrow) in the arterial phase (**b**); while in the nephrographic phase (**c**) the parenchymal contusion is more clearly recognized and the pseudoaneurysm is less visible. B-mode US (**d**) showed inhomogeneous echogenic areas (asterisks) corresponding to the contusions; CEUS (**e**) adequately showed all the vascular phases of the pseudoaneurysm (curved arrow) and the perirenal hematoma (asterisk); CEUS follow-up performed on 7th day (**f**). *Image source: Granata A, Campo I, Lentini P, Pesce F, Gesualdo L, Basile A, et al. Role of Contrast-Enhanced Ultrasound (CEUS) in Native Kidney Pathology: Limits and Fields of Action. Diagnostics. 2021 Jun 8;11(6):1058. This is an open access article distributed under the terms of the Creative Commons Attribution (CC BY) 4.0 license. (http://creativecommons.org/licenses/by/4.0/)

The most common pseudotumours are hypertrophic columns and fetal lobulation, which are quite common. These two conditions can mimic a solid lesion, especially for less experienced radiologists. Hypertrophic columns will appear on B-mode US as nodular lesions protruding into the renal sinus. Doppler can help in these cases by distinguishing between solid malignant lesions and anatomical variants as there is no distortion of the renal vascular architecture on Doppler, though making such distinctions requires radiological expertise. CEUS, however, is more immediate because it shows that the enhancement of these nodules is identical to that of the surrounding parenchyma. The high capacity of CEUS to detect enhancement, even for small lesions, makes it an excellent method for identifying contrast in suspicious lesions with pseudo-enhancement on CT [1, 10] (Figs. 1, 2, 3, 4, 5).

#### Follow-up of renal lesions under active surveillance

This is another indication for CEUS according to EFSUMB guidelines. Most renal lesions are detected incidentally during exams performed for other reasons, and this incidental detection has exponentially increased over the past few decades [1, 10]. Renal cancer was previously considered the internist's tumour, presenting as a palpable mass, flank pain, and haematuria, but it is now seen as the radiologist's tumour as we are responsible for most initial diagnoses. More frequently, we are detecting small renal masses (less than 4 cm), which are benign in 25% of cases and aggressive lesions (renal cell carcinoma, RCC) in 10%, with a metastasis risk below 2%. The likelihood of benignity increases as the mass size decreases [11–13]. More often, these lesions are found in elderly patients over seventy with comorbidities, raising issues regarding differential diagnosis and patient management. Once a lesion is detected, the patient must be directed to a diagnostic and therapeutic pathway. For all these reasons, over the past 10 years there has been growing advocacy for active surveillance rather than immediate surgical intervention for these small renal lesions. Active surveillance involves monitoring tumour size through imaging (US, CT, or MRI). Active surveillance differs from watchful waiting, which is reserved for patients whose comorbidities contraindicate any further active treatment and do not require imaging monitoring unless clinically indicated [14]. However, there are currently no specific guidelines regarding the timing of active surveillance or the methods to be used, though a method with low biological and economic cost should be employed. The EAU guidelines state that the lesions to be subjected to active surveillance are numerous as they represent the majority of those detected daily (Table 2). Given these challenges, the role of CEUS in the follow-up of small renal masses has gained increasing attention. CEUS provides real-time dynamic assessment of lesion enhancement, which is crucial for differentiating between benign and malignant renal masses. Lesions that show homogeneous hyperenhancement with slow washout are often indicative of benign conditions, such as oncocytomas or angiomyolipomas, making them suitable for active surveillance. Conversely, lesions demonstrating rapid washout, rim-like enhancement, or irregular vascularization raise suspicion for RCC and may necessitate early therapeutic intervention. In addition, CEUS has proven valuable in assessing lesion perfusion over time. For example, a growing but persistently hypo-enhancing lesion with minimal vascularity may suggest a low-grade tumour with indolent behaviour, supporting continued surveillance. On the other hand, the development of necrotic areas or increasing vascular heterogeneity could signal tumour progression, prompting the need for biopsy or definitive treatment. Given its ability to detect subtle perfusion changes, integrating CEUS into active surveillance protocols may improve risk stratification and clinical decision-making. The ACR has included CEUS as a suitable method for the follow-up of lesions under active surveillance, with appropriateness comparable to CT or MRI, as it is a method free from ionizing radiation, uses a non-nephrotoxic contrast agent, and is also cost-effective. Recent studies such as that of Aaron Oh et al. have demonstrated that CEUS is more cost-effective than traditional imaging techniques such as CT or MRI in the active surveillance of Bosniak III and IIF complex renal masses [15].

**Table 2:** Factors influencing active surveillance of renal lesions

Patient-related factors	Tumour-related factors
– Elderly – Life expectancy < 5 years – High comorbidities – Excessive perioperative risk – Frailty (poor functional status) – Patient preference for AS – Marginal renal function	– Renal cell carcinoma – Tumour size < 3 cm – Tumour growth < 5 mm/year – Non-infiltrative – Low complexity – Favourable histology – Predominantly cystic

#### Characterization of indeterminate lesions in patients with transplanted kidneys

These patients are inherently nephropathic as they have only one functioning kidney, and they often reject iodinated contrast agents due to the fear of side effects, particularly those associated with renal excretion. CEUS, having pulmonary excretion, is extremely valuable in this context.

When evaluating the transplanted kidney, it is equally important to assess the native kidney of the transplanted patient. Especially in patients who underwent dialysis prior to transplantation, native kidneys are prone to a condition called acquired cystic kidney disease (ACKD), which increases the risk of developing renal cancer by 19% compared to the general population. Thus, it is crucial to implement surveillance, particularly of the native kidney, which is often done with US alone [6]. If CEUS raises the suspicion of a renal lesion, the patient should be referred for CT or MRI, especially to guide surgical planning. In the case of equivocal lesions on B-mode US, it is advisable to perform CEUS to determine whether the lesion is vascularized or not. If the lesion is not vascularized, the patient may avoid undergoing a CT scan that uses nephrotoxic contrast agents.

#### Inflammatory-infectious pathologies

**Fig. 6:**
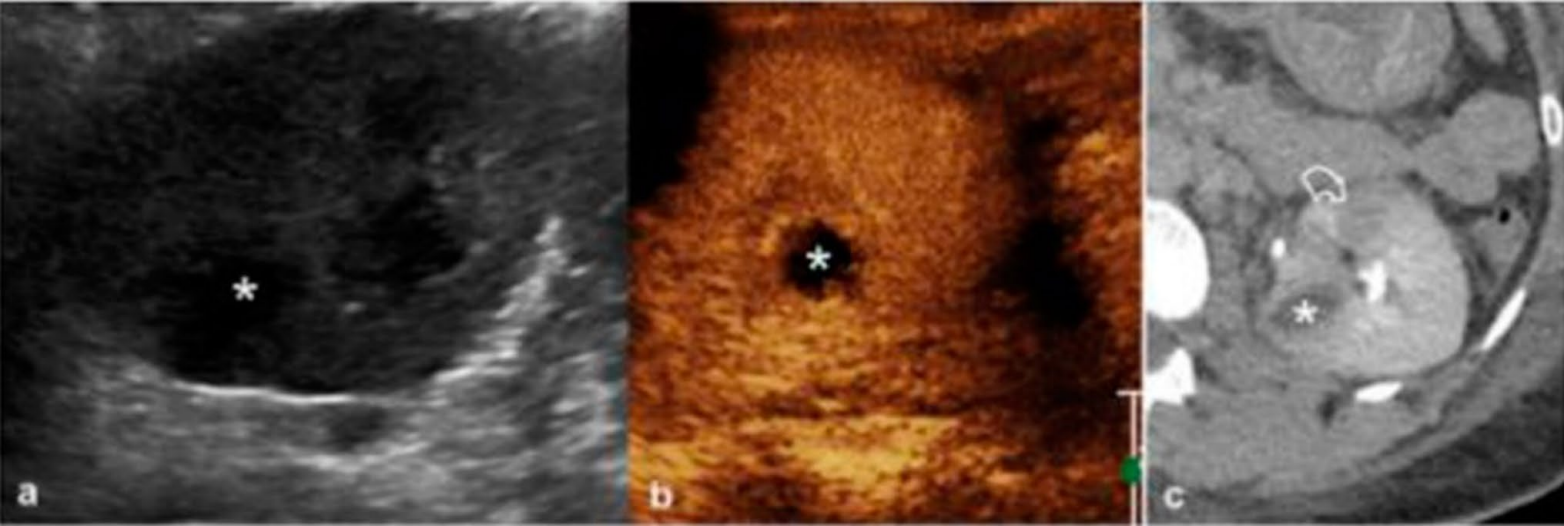
**a-c** A case of complicated pyelonephritis. US images of a renal evaluation in B-mode (**a**) showed an inhomogeneous, hypoechoic area (asterisk). CEUS (**b**) confirmed the presence of an avascular lesion with a peripheral rim, consistent with abscess (asterisk). Urographic phase CECT (**c**) confirmed the presence of the abscess (asterisk) and of a hypo-perfused area (curved arrow) at the lower pole of the kidney. *Image source: Granata A, Campo I, Lentini P, Pesce F, Gesualdo L, Basile A, et al. Role of Contrast-Enhanced Ultrasound (CEUS) in Native Kidney Pathology: Limits and Fields of Action. Diagnostics. 2021 Jun 8;11(6):1058. This is an open access article distributed under the terms of the Creative Commons Attribution (CC BY) 4.0 license. (http://creativecommons.org/licenses/by/4.0/)

Among the indications for CEUS are inflammatory-infectious pathologies, such as pyelonephritis. CEUS can detect the presence of acute pyelonephritis, which appears as areas of poorly vascularized parenchyma. In the acute phase, these areas tend to have a more rounded shape, but over time, they acquire a triangular morphology. Renal abscesses are also easily visible on CEUS, even when small in size, presenting as rounded lesions without contrast enhancement, sometimes with a rim of perilesional enhancement. The diagnostic accuracy of CEUS appears to be lower in cases of diffuse pyelonephritis due to the inability to simultaneously compare both kidneys. However, in focal and multifocal forms, authors have reported significant diagnostic accuracy, with a positive predictive value of 100% and a negative predictive value of 89%, though this was observed in a selected population with a high probability of disease [16, 17]. In addition to diagnosis, CEUS is important for monitoring the evolution of abscessed lesions. It allows for detailed measurement of lesion size and, more importantly, the precise differentiation between liquefied and peripheral areas. Focal pyelonephritis often appears on gray-scale US as a focal hyperechoic lesion with cortical swelling. The hyper-echogenicity on B-mode is due to numerous interfaces correlated with abundant inflammatory cells in the interstitium and tubules. In addition to the inflammatory infiltrate and edema, severe capillary damage has been demonstrated, with the presence of intraluminal leukocytes and fibrin plugs. These pathological findings correlate with the hypo-echogenic appearance on CEUS and the reduced enhancement of these lesions compared to the surrounding cortex. Areas of focal pyelonephritis are more visible during the late parenchymal phase as these wedge-shaped or rounded areas appear more hypo-echogenic during this phase. Abscesses appear as heterogeneous or hypo-echogenic focal lesions with cortical swelling. CEUS can be used to assess the size, internal structure, and extent of abscesses. An abscess is a necrotic cavity containing pus and debris that does not exhibit enhancement during any phase. Conversely, enhancement of the septa can be seen if the abscess is partially liquefied. Enhancement of the peripheral rim can also be observed during the cortical phase. Focal pyelonephritis and abscesses of various sizes appear in different phases of CEUS. Abscesses are characterized by a rounded shape and an anechoic structure on B-mode. They are completely avascular in all post-contrast phases and may have a thin rim of enhancement. Areas of pyelonephritis are generally located in the cortical region and are typically isoechoic or hyperechoic on B-mode, wedge-shaped, and appear hypo-vascular after contrast injection. They are more evident during the late parenchymal phase (Fig. 6).

#### Additional roles of CEUS in solid mass lesion evaluation

One of the indications from EFSUMB guidelines includes the study of advanced renal lesions to evaluate caval thrombosis, not just the renal lesion itself. Studies have demonstrated the efficacy of CEUS in assessing thrombosis, distinguishing between neoplastic and non-neoplastic thrombi [18, 19]. CEUS is useful for determining whether a thrombus shows post-contrast enhancement, which is critical information for surgeons when deciding if the thrombus is a red thrombus or a neoplastic thrombus. If it is a neoplastic thrombus, it will exhibit contrast enhancement. Another important application of CEUS, as outlined by EFSUMB guidelines, is the evaluation of solid lesions that may develop in the surgical bed following nephrectomy. Studies have provided evidence of CEUS aiding in the differentiation of recurrent tumours from post-surgical fibrosis. This differentiation is crucial for guiding further treatment decisions, including the need for additional surgical intervention or targeted therapy [20].

## Conclusion

In conclusion, CEUS, due to its excellent spatial and temporal resolution, can be useful for differential diagnosis in uncertain cases, particularly in differentiating between pseudotumours, solid tumours, and cystic tumours, and may increase confidence in clinical decision-making. Additionally, CEUS serves as an imaging modality for some patients when CT or MR cannot be performed. Furthermore, CEUS can be used for follow-up, monitoring, and evaluating the efficacy of treatment methods. However, for now, it is more frequently used as a complementary method to other imaging modalities in the evaluation of renal lesions.

### Highlights

- CEUS enhances renal lesion diagnosis by providing detailed vascularization information, surpassing the limitations of B-mode ultrasound in distinguishing solid from cystic masses and identifying malignancies.
- It uses a safe, non-nephrotoxic contrast agent suitable for patients with kidney impairment, requires minimal resources, and is cost-effective and widely accessible.
- EFSUMB guidelines highlight its applications for characterizing indeterminate renal lesions, monitoring non-surgical cases, diagnosing infections, and evaluating advanced renal lesions and surgical bed masses.
- CEUS is especially beneficial for patients in whom CT or MRI is contraindicated, offering high sensitivity in assessing microcirculation and creating advanced imaging maps for lesion follow-up.
- Despite technical limitations, such as deep lesion visualization and off-label regulatory status, CEUS is a valuable complementary tool to other imaging modalities like CT and MRI.
